# Bone imaging modality precision and agreement between DXA, pQCT, and HR-pQCT

**DOI:** 10.1093/jbmrpl/ziae158

**Published:** 2024-12-03

**Authors:** Jakub Mesinovic, Mícheál Ó Breasail, Lauren A Burt, Cat Shore-Lorenti, Roger Zebaze, Camelia Q E Lim, Zihui Ling, Peter R Ebeling, David Scott, Ayse Zengin

**Affiliations:** Institute for Physical Activity and Nutrition (IPAN), School of Exercise and Nutrition Sciences, Deakin University, Burwood, VIC 3125, Australia; Department of Medicine, School of Clinical Sciences at Monash Health, Monash University, Clayton, VIC 3168, Australia; Department of Medicine, School of Clinical Sciences at Monash Health, Monash University, Clayton, VIC 3168, Australia; Population Health Sciences, Bristol Medical School, University of Bristol, United Kingdom; McCaig Institute for Bone and Joint Health, Cumming School of Medicine, University of Calgary, Calgary AB, Canada; Department of Medicine, School of Clinical Sciences at Monash Health, Monash University, Clayton, VIC 3168, Australia; Department of Medicine, School of Clinical Sciences at Monash Health, Monash University, Clayton, VIC 3168, Australia; Department of Medicine, School of Clinical Sciences at Monash Health, Monash University, Clayton, VIC 3168, Australia; Department of Medicine, School of Clinical Sciences at Monash Health, Monash University, Clayton, VIC 3168, Australia; Department of Medicine, School of Clinical Sciences at Monash Health, Monash University, Clayton, VIC 3168, Australia; Institute for Physical Activity and Nutrition (IPAN), School of Exercise and Nutrition Sciences, Deakin University, Burwood, VIC 3125, Australia; Department of Medicine, School of Clinical Sciences at Monash Health, Monash University, Clayton, VIC 3168, Australia; Department of Medicine, School of Clinical Sciences at Monash Health, Monash University, Clayton, VIC 3168, Australia

**Keywords:** bone QCT/microCT, analysis/quantitation of bone, DXA, analysis/quantitation of bone, radiology, statistical methods, fracture prevention, HR-pQCT, pQCT, precision

## Abstract

Quantifying precision error for DXA, peripheral QCT (pQCT), and HR-pQCT is crucial for monitoring longitudinal changes in body composition and musculoskeletal outcomes. Agreement and associations between bone variables assessed using pQCT and second-generation HR-pQCT are unclear. This study aimed to determine the precision of, and agreement and associations between, bone variables assessed via DXA, pQCT, and second-generation HR-pQCT. Thirty older adults (mean age: 64.2 **±** 8.0 yr; women: 67%) were recruited. DXA scans were performed at the total hip, lumbar spine, and whole body. Distal (4%) and proximal (30%/33%/66%) skeletal sites at the radius and tibia were scanned with pQCT and/or HR-pQCT. Root-mean-squared coefficients of variation (%CV_RMS_) were calculated to define precision errors, and Bland–Altman plots assessed agreement between densitometric estimates. Pearson correlations and linear regression explored relationships between bone variables at different skeletal sites and proportional bias, respectively. Precision errors ranged between 0.55% and 1.6% for DXA, 0.40% and 4.8% for pQCT, and 0.13% and 30.7% for HR-pQCT. Systematic bias was identified between pQCT- and HR-pQCT-determined radius and tibia volumetric BMD (vBMD) estimates (all *p*<.001). Proportional bias was not observed between vBMD measures at any skeletal site (all *p*>.05). pQCT- and HR-pQCT-determined total, trabecular, and cortical vBMD and estimates of bone strength at the radius and tibia were strongly correlated (all *p*<.05). Precision error was low for most bone variables and within the expected range for all imaging modalities. We observed significant systematic bias, but no proportional bias, between pQCT- and second-generation HR-pQCT-determined vBMD estimates at the radius and tibia. Nevertheless, measures of bone density and strength were strongly correlated at all skeletal sites. These findings suggest that although bone density and strength estimates from both imaging modalities are not interchangeable, they are strongly related and likely have similar fracture prediction capabilities.

## Introduction

A limitation shared by all quantitative imaging modalities is the degree of inherent random error that exists between serial scans, known as precision error, which exists even when all variables (eg, same patient, operator, and scan site) are kept constant.^1^ Precision error is exacerbated by patient repositioning between scans.^1^ Despite major advances in bone imaging in recent decades, precision error still presents a major challenge to ensuring densitometry results provide accurate and reproducible assessments of rates of change and reliable cross-sectional comparisons.^1^ Accurate imaging assessments rely on awareness of the least significant change (LSC) determined at the facility where measurements occur. If measured changes exceed the LSC, confident therapeutic decisions can be made.^1^ This standardized approach prevents misinterpretation of random variability, ensuring effective therapeutic regimens are maintained and unnecessary interventions are avoided, ultimately optimizing patient care.^1^^,^^2^

In research and clinical settings, DXA, a 2D imaging modality, is the gold standard for densitometry and most used.^3^ However, a major limitation of DXA is that it is incapable of providing compartment-specific measures of bone. Peripheral QCT (pQCT) technologies are 3D imaging modalities that provide compartment-specific estimates of density, mass, geometry, distribution, and (micro)structure.^4^ The resolution of single-slice pQCT scanners varies with a voxel size of 0.2-0.8 mm, whereas the latest second-generation HR-pQCT scanners have a voxel size of approximately 61 μm.^4^ Smaller voxel size (ie, higher resolution) offers advantages, including the ability to directly assess bone microstructure; however, voxel size differences may affect the comparability of estimates obtained via older and newer pQCT technologies. At testing sites where patients are monitored over long periods, it is important to understand how scan resolution influences comparability, given imaging modalities are constantly evolving. Only two studies have explored agreement and associations between bone variables assessed by pQCT and HR-pQCT,^5^^,^^6^ and one was performed ex vivo.^5^ Both studies reported relatively poor agreement, but strong correlations, between the same bone variables measured on both imaging modalities.^5^^,^^6^

Short-term precision has been reported in several studies for DXA,^7–9^ pQCT,^10–12^ and second-generation HR-pQCT.^13–15^ Generally, DXA has lower precision error due to the larger scanning regions and shorter scan times, and it is less sensitive to both operator positioning and participant movement. Standard HR-pQCT precision is based on 2D registration (2DR) involving areal slice matching. Under ideal conditions, participants would be positioned and scanned identically at all time points; however, in reality, this is seldom the case, and small discrepancies in positioning can lead to bias. Novel approaches for improving HR-pQCT precision in longitudinal studies have emerged in recent years, including 3D registration (3DR) and matched angle (MA) image registration.^16–18^ 3DR mitigates positioning errors that manifest as 3D misalignment (ie, rotations and translations) between serial images, leading to better reproducibility for bone density and microarchitecture compared with 2DR.^16^^,^^18–20^

The standard offsets used for HR-pQCT, which differ by limb and scanner generation, are defined based on fixed distances from the endplate; however, in more recent years the use of relative offsets has become more common.^21^ This approach is consistent with what has been standard practice with single-slice pQCT for decades; the justification for which is based on the fact that fixed-distance scan sites may not be equivalent in bone composition between individuals with different limb lengths.^22^ Only one in vivo study^17^ has compared 2DR and 3DR using relative offsets at the radius and tibia, which differ in terms of densitometry and (micro)structure compared with the manufacturers’ fixed offset; this may influence precision estimates.^22^ The study^17^ reported improved precision following 3DR, but they only recruited adults with osteogenesis imperfecta, so it is unclear to what extent 3DR influences precision estimates of bone variables measured using relative offsets in healthy adults.

This study aimed to: (1) report short-term precision estimates for bone and body composition measures at our scanning facility with DXA, pQCT, and HR-pQCT; (2) explore whether 3DR improves precision estimates from HR-pQCT scans at fixed and relative offsets; (3) determine agreement between pQCT and HR-pQCT densitometric estimates; and (4) explore associations between bone density and strength estimates determined by pQCT and HR-pQCT.

## Methods

### Study design

In total, 30 adults aged *>*50 yr were recruited between November 2019 and March 2020 via various methods, including advertisement of posters at Monash Medical Centre, local general practitioners, community centers, businesses, and Monash University. Prospective study participants completed a standard screening questionnaire, and the data was used to select individuals for inclusion in the study. Inclusion criteria included adults who were fluent in written and spoken English, had a body mass <160 kg, had at least one upper- and lower-limb free from metal or other material, and had no medical conditions that would prevent them from laying still or in a supine position during scans. Exclusion criteria were pregnancy or recent exposure to nuclear medicine. This study was conducted at a single site (Monash Health Translational Research Facility, Melbourne, Australia) according to the principles of the Declaration of Helsinki and approved by the Monash Health Human Research Ethics Committee (Protocol ID: RES-18-0000-508A). All participants provided written informed consent.

This study adhered to the guidelines listed in the 2016 Official Positions of the International Society of Bone Densitometry.^2^ We recruited 30 participants and performed two scans on all participants at each skeletal site of interest for each imaging modality. The precision scan procedure for each participant per manufacturer recommendations was as follows: (1) initialize imaging modality, (2) position participant, (3) scan participant (scan one), (4) repeat steps 1-3 (scan two), and (5) proceed to next scan site and/or imaging modality and repeat steps 1-4. All scans were performed on the same day, and participants had a 15-min break before undertaking scans on a different imaging modality.

### Questionnaires and anthropometric measurements

Participants completed questionnaires about general demographic information, medication usage, family history of fractures, and chronic health conditions.

Weight was measured to the nearest 0.1 kg using electronic scales (Seca 804, Seca), and height was measured to the nearest 0.1 cm using a wall-mounted stadiometer (Seca 213, Seca) after footwear and heavy items of clothing were removed. BMI was calculated as weight (kg)/height (m^2^).

### Dual-energy X-ray absorptiometry

Participants underwent whole-body DXA scans (Hologic Discovery A, Hologic) for assessment of body composition (whole-body lean and fat mass [g], appendicular lean mass [g], and visceral adipose tissue [cm^2^]) and whole-body areal BMD (aBMD; g/cm^2^). Scans were also performed at the total hip and anteroposterior lumbar spine to assess aBMD (g/cm^2^). The total effective dose for all DXA scans was approximately 0.024 mSv. Scans were acquired and analyzed by one investigator (A.Z. and C.S.L., respectively) using the manufacturer’s standard protocol and software (Hologic Apex version 5.6.0.2).

### Peripheral quantitative computed tomography

Scans were acquired with pQCT (Stratec XCT3000, Stratec) by one investigator (D.S.) in the non-dominant forearm (radius) and lower leg (tibia). Participants were seated with their forearm or lower leg positioned inside the pQCT gantry. A single 2.5 mm transverse scan was acquired with an in-plane voxel size of 0.5 mm, a scan speed of 20 mm/s, an X-ray tube voltage of 61 kV, and an X-ray tube current of 0.229 mA. Radius length was measured from the distal end of the ulna styloid to the tip of the olecranon; tibia length was measured from the end of the medial malleolus to the tibial plateau. Scan sites were determined using a planar scout view (scout scan speed: 30 mm/s) of the distal radius or tibia, and reference lines were placed to bisect the medial border of the distal radial joint surface and parallel to the distal joint surface of the tibia. Scans were acquired at 4%, 33%, and 66% of the radius and 4%, 38%, and 66% of the tibia. The total effective dose for all scans was approximately 0.005 mSv.

All images were analyzed by one investigator (M.O.B.) using the manufacturer’s software (version 6.2). At the distal sites (4%), CALCBD with contour mode 1 and a threshold of 180 mg/cm^3^ was used to calculate total cross-sectional area (CSA; mm^2^), as well as total and trabecular volumetric BMD (vBMD; mg/cm^3^). Peel mode 1 used a concentric peel to remove the outer 55% of the total CSA, designating the remaining inner 45% CSA as trabecular bone. At proximal sites (33%/38%/66%), CORTBD with separation mode 1 and a threshold of 710 mg/cm^3^ was used to define cortical vBMD, cortical CSA, and cortical bone mineral content (BMC; mg/mm), while total CSA was determined using an edge detection threshold of 280 mg/cm^3^. Cortical thickness (mm) was calculated using the circular ring model.^23^ Polar strength-strain index (SSI polar; mm^3^), polar moment of inertia based on cortical area (pMOIa; mm^4^), and polar moment of inertia based on cortical area weighted by cortical density (pMOIw; mm^4^), which are estimates of torsional bone strength, were obtained at a threshold of 280 mg/cm^3^ using cortmode 1. Body composition variables were also obtained from the proximal lower limb. Muscle CSA (mm^2^) was calculated by subtracting the combined bone area of the tibia and fibula (threshold 280 mg/cm^3^) from muscle and bone CSA (threshold 40 mg/cm^3^). Muscle density (mg/cm^3^) was obtained using a smoothing filter (F03F05) at a threshold of 100 mg/cm^3^. We used a 4-point scan grading scale to categorize motion artifacts in scans. A score of 1 was considered perfect; 2 was considered good with very minor amounts of motion artifact; 3 was assigned to scans where bone data was salvageable but muscle data was not; 4 was given to unusable scans, which were excluded from analyses. The number of scans excluded at different skeletal sites was as follows: radius *n*: 4% = 1, 33% = 5, 66% = 9; tibia *n*: 4% and 38% = 1, 66% = 1 except for muscle CSA (*n* = 3). The manufacturer’s phantom was scanned on all data collection days for quality control.

### High-resolution quantitative computed tomography

Scans with HR-pQCT (XtremeCT II, Scanco Medical) were acquired by one investigator (C.S.L.) in the non-dominant forearm (radius) and lower leg (tibia). Non-dominant limbs were placed in a cast and stabilized to prevent movement artifacts. Scan sites were determined using a planar scout view of the distal radius or tibia, and reference lines were placed to bisect the medial border of the distal radial joint surface and parallel to the distal joint surface of the tibia. Scans were acquired 9.0 and 22.0 mm proximal from the reference line for the radius and tibia (fixed offset), respectively, as per the standard protocol.^21^ Scans were also acquired at 4% of the radius and 30% of the tibia. One hundred and sixty-eight parallel slices encompassing a 10.197 mm region were acquired at each scan site at the radius and tibia. Scans had a voxel size of 60.7 μm, X-ray tube voltage was 68 kVp, and X-ray tube current was 1470 μA. The total effective dose for all scans was approximately 0.030 mSv.

HR-pQCT scans were analyzed using the manufacturer’s standard protocol by three investigators (C.S.L., C.Q.L., and Z.L.), which involves slice-matching scans based on a 2D cross-sectional area (Image Processing Language, v5.42, Scanco Medical AG). To further improve precision, we additionally performed 3DR of scans to define a consistent volume of interest (VOI).^20^ Briefly, a whole bone mask was used to identify the region to sample image data and the second scan acquired from each participant was registered to their first scan to ensure a consistent volume of interest. To initialize the registration, the centers of mass in the masked regions of scans two and one were aligned. All morphometric and density variables were determined in the scan two image space within the largest common volume defined by the common mask. To quantify repositioning error with 3DR, the rotation angle between the longitudinal axes of scans one and two were calculated. For all registration techniques, the percent overlap was defined as the common mask volume divided by the total volume in scan one. The overlap between HR-pQCT scan volumes included in 3D registration was >90% between scan pairs, ie, at least 150 common slices would be shared between both 168-slice stacks. Bone variables from standard HR-pQCT analyses included: total, trabecular, and cortical vBMD (mg/cm^3^); cortical porosity (%, the segmented pore volume); cortical thickness (mm); trabecular thickness (mm); trabecular separation (mm, the mean space between trabeculae); trabecular number (1/mm); and trabecular bone volume fraction (BV/TV; %). The manufacturer’s bone midshaft evaluation script was used with an outer threshold of 450 mgHA/cm^3^ and a low-pass Gaussian filter (sigma 0.8, support 1.0) to estimate the polar moment of inertia (pMOI; mm^4^) of the whole bone.^24^ We also performed finite element analysis (FEA) at all sites to estimate bone stiffness (kN/mm) and failure load (kN). While most HR-pQCT variables were assessed using 3DR, it was not performed on measures of bone strength derived via FEA. This was due to the added difficulty of dealing with the complex (ie, non-parallel) proximal and distal surfaces of the common region identified using this technique. We used a 5-point grading scale^25^ to categorize motion artifacts in scans. Scans graded 5 were excluded from all analyses, while those graded 3 or lower were included (excluded scans: radius 4% *n* = 1; tibia fixed offset *n* = 1).

### Statistical analyses

Descriptive data are expressed as mean (95% confidence intervals) or frequency (%). We assessed the precision errors of DXA-, pQCT-, and HR-pQCT-bone variables by calculating root-mean-squared SD (SD_RMS_) and root-mean-squared coefficients of variation (%CV_RMS_) for each outcome using the following equations:


(1) \begin{equation*} S{D}_{RMS}=\sqrt{\frac{\sum_{i=1}^m\left(S{D}_{Pm}^2\right)}{m}} \end{equation*}

where SD_Pm_ is the average SD for all participants and *m* is the number of participants.


(2) \begin{equation*} C{V}_{RMS}=\sqrt{\frac{\sum_{i=1}^m\left(C{V}_{Pm}^2\right)}{m}} \end{equation*}

where CV_Pm_ is the average coefficient of variation for all participants and *m* is the number of participants.

Bland–Altman plots^26^ were used to calculate agreement between densitometric estimates from pQCT and HR-pQCT; scan one was used in these analyses. One-sample *t*-tests were used to assess systematic bias, and linear regression was used to determine proportional bias. Pearson correlations explored associations between densitometric and strength estimates from different imaging modalities. Agreement and correlations between pQCT and HR-pQCT variables were performed at the closest comparable site for each limb. Bland–Altman and scatter plots were created using GraphPad Prism 10 (San Diego, CA). *p* values <.05 or 95% confidence intervals (CIs) not including the null point were considered sufficient to reject the null hypothesis.

## Results

Descriptive characteristics of the entire cohort are presented in Table 1. Participants had a mean age of 64.2 yr (range: 51-81 yr), body mass of 72.6 kg, and BMI of 27.3 kg/m^2^. Most participants were women (*n* = 20; 67%), almost a quarter had self-reported osteoporosis (*n* = 7; 23%), and 20% (*n* = 6) used osteoporosis medications.

**Table 1:** Descriptive characteristics.

	**Mean (95%CI) or frequency (%)**
**Age (yr)**	65.5 (62.5, 68.5)
**Women**	20 (66)
**Body mass (kg)**	72.6 (67.7, 77.5)
**Height (cm)**	162.7 (159.6, 165.9)
**Body mass index (kg/m^2^)**	27.3 (25.7, 28.9)
**Self-reported osteoporosis**	7 (23)
**Family history of hip fracture**	5 (17)
**Self-reported osteoporosis medication usage**	6 (20)

Tables 2 and 3 present short-term precision data from DXA and pQCT at different skeletal sites. The CV for the total hip, femoral neck, and lumbar spine aBMD measured by DXA was 0.55%, 1.60%, and 0.90%, respectively. Whole-body fat and lean mass had CVs *≤*0.96%. For pQCT at the distal radius, CVs for total CSA and vBMD and trabecular vBMD were *≤*4.44%. CVs at the proximal radius were lowest for vBMD estimates (range: 0.58%-0.85%) and highest for estimates of bone geometry and strength. At the distal tibia, CVs for total CSA and vBMD and trabecular vBMD were *≤*2.43%. CVs at the proximal tibia were lowest for densitometric estimates (all approximately 0.40%) and highest for estimates of bone geometry and strength and body composition (66% muscle CSA: 5.89%; 66% muscle density: 0.97%).

**Table 2:** Short-term DXA precision for total hip, lumbar spine, and areal BMD and fat and lean mass.

	**SD**_**RMS**_	**%CV**_**RMS**_
**Total hip aBMD (g/cm^2^)**	0.005	0.55
**Femoral neck aBMD (g/cm^2^)**	0.012	1.60
**Trochanteric aBMD (g/cm^2^)**	0.008	1.09
**Intertrochanteric aBMD (g/cm^2^)**	0.009	0.84
**Lumbar spine aBMD (g/cm^2^)**	0.010	0.90
**Whole body aBMD (g/cm^2^)**	0.008	0.82
**Whole body total fat mass (g)**	262.871	0.96
**Visceral adipose tissue (cm^2^)**	6.112	4.74
**Whole body total lean mass (g)**	268.933	0.57
**Appendicular lean mass (g)**	172.117	0.90

Abbreviation: aBMD, areal BMD.

**Table 3:** Short-term pQCT precision at the distal and proximal radius and tibia.

	**Radius**			**Tibia**		
	**n**	**SD**_**RMS**_	**%CV**_**RMS**_	**n**	**SD**_**RMS**_	**%CV**_**RMS**_
**4% site**						
** Total CSA (mm^2^)**	29	19.650	4.44	29	10.386	0.87
** Total vBMD (mg/cm^3^)**	29	10.062	4.00	29	4.065	1.22
** Trabecular vBMD (mg/cm^3^)**	29	4.788	2.36	29	8.192	2.43
**33/38% site**						
** Total CSA (mm^2^)**	25	1.645	1.37	29	13.360	1.28
** Cortical CSA (mm^2^)**	25	1.165	1.43	29	3.333	1.17
** Cortical BMC (mg/mm)**	25	1.269	1.30	29	2.975	0.99
** Cortical vBMD (mg/cm^3^)**	25	6.727	0.58	29	4.603	0.40
** Cortical thickness (mm)**	25	0.037	1.38	29	0.070	1.39
** SSI (mm^3^)**	25	6.332	2.45	29	27.399	1.79
**66% site**						
** Total CSA (mm^2^)**	21	2.986	2.20	29	4.915	0.79
** Cortical CSA (mm^2^)**	21	1.813	2.84	29	3.838	1.45
** Cortical BMC (mg/mm)**	21	2.007	2.92	29	4.328	1.47
** Cortical vBMD (mg/cm^3^)**	21	9.382	0.85	29	4.498	0.41
** Cortical thickness (mm)**	21	0.048	2.62	29	0.105	2.70
** SSI (mm^3^)**	21	7.411	2.92	29	84.6	3.29
** Muscle density (mg/cm^3^)**	–	–	–	29	0.650	0.97
** Muscle CSA (mm^2^)**	–	–	–	27	376.7	5.89

Radius scanned at 33%; tibia scanned at 38%.

Abbreviations: BMC, bone mineral content; CSA, cross-sectional area; SSI, strength strain index; vBMD, volumetric BMD.

HR-pQCT short-term precision at the radius is presented in Table 4. Only one set of 3D registered scans had <90% overlap (87%; fixed offset). Using 2DR, CVs were lowest for vBMD estimates (range: 0.34%-0.84%) and highest for estimates of cortical porosity (range: 14.08%-30.70%) at all skeletal sites. At the fixed site, CVs ranged between 0.82% and 3.20% for trabecular and cortical microarchitecture variables and were approximately 4% for bone stiffness and failure load. At the 4% site, CVs for trabecular microarchitecture variables were lowest for trabecular thickness (1.10%) and highest for trabecular number (2.51%), while CVs for bone stiffness and failure load were approximately 7%. At the 30% site, the CV for cortical thickness was 1.62%, and CVs for bone stiffness and failure load were *<*0.26%. After scans underwent 3DR, the precision for most variables improved (generally by <1%) except for trabecular number at the 4% site and cortical vBMD and porosity at the fixed and 4% sites, respectively.

**Table 4:** Short-term HR-pQCT precision at the radius using 2D and 3D registration.

	**2D registration**		**3D registration**		
	**SD**_**RMS**_	**%CV**_**RMS**_	**SD**_**RMS**_	**%CV**_**RMS**_	**2D CV – 3D CV**
**Fixed site (9.0 mm offset)**					
** Total vBMD (mg HA/cm^3^)**	1.156	0.39	0.879	0.29	0.10
** Trabecular vBMD (mg HA/cm^3^)**	0.745	0.59	0.645	0.51	0.08
** Trabecular number (1/mm)**	0.047	3.20	0.047	3.14	0.06
** Trabecular separation (mm)**	0.018	2.19	0.016	2.11	0.08
** Trabecular thickness (mm)**	0.002	0.93	0.002	0.92	0.01
** Trabecular BV/TV (%)**	0.002	1.09	0.002	1.00	0.09
** Cortical vBMD (mg HA/cm^3^)**	4.590	0.53	4.810	0.56	−0.03
** Cortical porosity (%)**	0.002	14.08	0.001	13.69	0.39
** Cortical thickness (mm)**	0.009	0.82	0.008	0.72	0.10
** Stiffness (kN/mm)**	2798.98	3.93	–	–	–
** Failure load (kN)**	154.47	4.01	–	–	–
**4% site** ^**a**^					
** Total vBMD (mg HA/cm^3^)**	1.855	0.81	1.376	0.59	0.22
** Trabecular vBMD (mg HA/cm^3^)**	1.093	0.84	0.888	0.68	0.16
** Trabecular CSA (mm^2^)**	0.606	0.21	1.155	0.37	−0.16
** Trabecular number (1/mm)**	0.037	2.51	0.038	2.54	−0.03
** Trabecular separation (mm)**	0.012	1.78	0.012	1.76	0.02
** Trabecular thickness (mm)**	0.003	1.10	0.003	1.10	0
** Trabecular BV/TV (%)**	0.003	1.58	0.244	1.43	0.15
** Cortical vBMD (mg HA/cm^3^)**	7.383	0.97	6.280	0.83	0.14
** Cortical CSA (mm^2^)**	0.626	1.11	0.363	0.71	0.40
** Cortical porosity (%)**	0.002	20.69	0.195	21.11	−0.42
** Cortical thickness (mm)**	0.008	1.13	0.007	0.97	0.16
** Stiffness (kN/mm)**	3073.03	7.27	–	–	–
** Failure load (kN)**	157.70	6.92	–	–	–
**30% site**					
** Cortical vBMD (mg HA/cm^3^)**	3.644	0.34	1.413	0.13	0.21
** Cortical porosity (%)**	0.002	30.7	0.002	11.21	19.49
** Cortical thickness (mm)**	0.051	1.62	0.009	0.37	1.25
** Stiffness (kN/mm)**	191.7	0.23	–	–	–
** Failure load (kN)**	13.55	0.26	–	–	–

a *n* = 29.

Abbreviations: BV/TV, bone volume fraction; CSA, cross-sectional area; vBMD, volumetric BMD.

Short-term precision at the tibia using HR-pQCT is presented in Table 5. All 3D-registered scans had >90% overlap. Using 2DR, CVs were lowest for vBMD estimates (range: 0.23%-0.82%) and highest for estimates of cortical porosity (range: 12.12%-15.25%) at all skeletal sites. At the fixed site, CVs ranged between 0.82% and 3.37% for trabecular and cortical microarchitecture variables. CVs for bone stiffness and failure load were *≤*2.83%. At the 4% site, CVs for trabecular microarchitecture variables were lowest for trabecular thickness (0.81%) and lowest for trabecular number (3.12%), while CVs for bone stiffness and failure load were *≤*7.3%. At the 30% site, the CV for cortical thickness was 2.06%, and CVs for bone stiffness and failure load were *≤*1.64%. After scans underwent 3DR, the precision of most variables improved (generally by <1%) except for trabecular thickness and number at the fixed and 4% site and trabecular vBMD and separation at the 4% site.

**Table 5:** Short-term HR-pQCT precision at the tibia using 2D and 3D registration.

	**2D registration**		**3D registration**		
	**SD**_**RMS**_	**%CV**_**RMS**_	**SD**_**RMS**_	**%CV**_**RMS**_	**2D CV – 3D CV**
**Fixed site** ^**a**^ **(22.0 mm offset)**					
** Total vBMD (mg HA/cm^3^)**	2.203	0.62	0.605	0.22	0.40
** Trabecular vBMD (mg HA/cm^3^)**	1.114	0.59	0.731	0.44	0.15
** Trabecular number (1/mm)**	0.045	3.37	0.045	3.42	−0.05
** Trabecular separation (mm)**	0.020	2.47	0.020	2.44	0.03
** Trabecular thickness (mm)**	0.003	0.97	0.003	0.97	0
** Trabecular BV/TV (%)**	0.002	0.82	0.002	0.72	0.10
** Cortical vBMD (mg HA/cm^3^)**	6.318	0.71	4.652	0.53	0.18
** Cortical porosity (%)**	0.004	12.12	0.004	11.70	0.42
** Cortical thickness (mm)**	0.022	1.34	0.014	0.91	0.43
** Stiffness (kN/mm)**	4088.74	2.83	–	–	–
** Failure load (kN)**	189.979	2.45	–	–	–
**4% site**					
** Total vBMD (mg HA/cm^3^)**	0.582	0.26	0.568	0.26	0
** Trabecular vBMD (mg HA/cm^3^)**	0.447	0.23	0.476	0.24	−0.01
** Trabecular CSA (mm^2^)**	0.851	0.08	1.434	0.16	−0.08
** Trabecular number (1/mm)**	0.056	3.12	0.056	3.14	−0.02
** Trabecular separation (mm)**	0.014	2.58	0.014	2.60	−0.02
** Trabecular thickness (mm)**	0.002	0.81	0.002	0.83	−0.02
** Trabecular BV/TV (%)**	0.002	0.85	0.204	0.82	0.03
** Cortical vBMD (mg HA/cm^3^)**	3.738	0.59	2.706	0.43	0.16
** Cortical CSA (mm^2^)**	0.935	1.15	0.603	0.74	0.41
** Cortical porosity (%)**	0.002	11.26	0.171	11.08	0.18
** Cortical thickness (mm)**	0.009	1.42	0.006	0.93	0.49
** Stiffness (kN/mm)**	9634.0	7.30	–	–	–
** Failure load (kN)**	464.5	5.79	–	–	–
**30% site**					
** Cortical vBMD (mg HA/cm^3^)**	8.029	0.82	7.275	0.74	0.08
** Cortical porosity (%)**	0.002	15.25	0.002	12.14	3.11
** Cortical thickness (mm)**	0.107	2.06	0.053	0.96	1.10
** Stiffness (kN/mm)**	1813.4	0.74	–	–	–
** Failure load (kN)**	222.7	1.64	–	–	–

a *n* = 29.

Abbreviations: BV/TV, bone volume fraction; CSA, cross-sectional area; vBMD, volumetric BMD.

**Figure 1:**
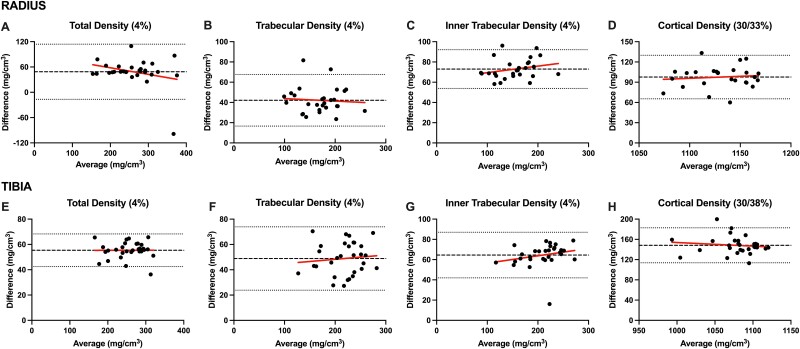
Bland–Altman plots of agreement between densitometric estimates from pQCT and HR-pQCT. (A-D) presents agreement for total (4% site), trabecular (4% site), inner trabecular (4% site), and cortical vBMD (pQCT: 33%; HR-pQCT: 30%) at the radius. (E-H) presents agreement for total (4% site), trabecular (4% site), inner trabecular (4% site), and cortical vBMD (pQCT: 38%; HR-pQCT: 30%) at the tibia. The X-axis represents the mean of the two measurements and the Y-axis represents the difference between the two measurements.

**Figure 2:**
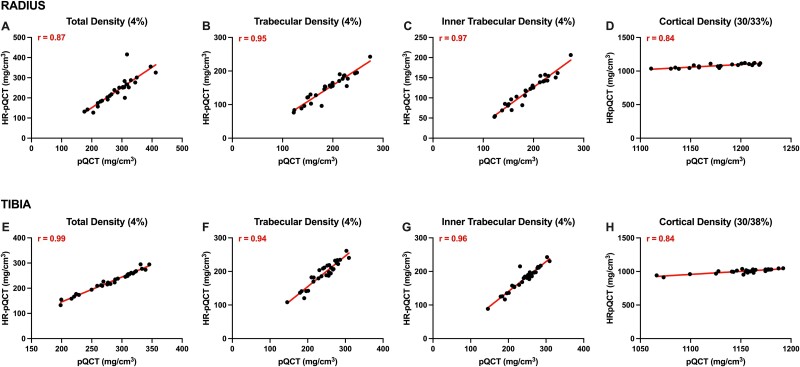
Pearson correlations between densitometric estimates from pQCT and HRpQCT. (A-D) presents associations for total (4% site), trabecular (4% site), inner trabecular (4% site), and cortical vBMD (pQCT: 33%; HRpQCT: 30%) at the radius. (E-H) presents associations for total (4% site), trabecular (4% site), inner trabecular (4% site), and cortical vBMD (pQCT: 38%; HRpQCT: 30%) at the tibia.

Bland–Altman plots of agreement and Pearson correlations between densitometric estimates from pQCT and HR-pQCT are presented in Figures 1 and 2. Densitometric estimates at all distal and proximal sites of the radius and tibia were higher with pQCT compared with HR-pQCT, resulting in systematic bias (all *p*<.001). Linear regression suggested no proportional bias (all *p*>.05) was evident for any densitometric estimates at the distal or proximal radius and tibia. Pearson correlations showed strong positive associations between densitometric estimates from pQCT and HR-pQCT at the distal and proximal radius and tibia (*r* = 0.84-0.99). HR-pQCT-derived failure load had strong negative correlations with SSI polar, pMOIa, and pMOIw (*r* = −0.90 to −0.94), and HR-pQCT-derived bone stiffness had strong positive correlations with pQCT-derived SSI polar, pMOIa, and pMOIw (*r* = 0.91-0.95) at the proximal radius and tibia; pMOI variables from both machines had moderate correlations at these skeletal sites (*r* = 0.52-0.65) (Table S1).

## Discussion

Precision error at our scanning facility was low and within the expected range for DXA, pQCT, and second-generation HR-pQCT. 3D registration reduced precision errors for most HR-pQCT-derived bone variables. We observed systematic bias, but no proportional bias, between pQCT- and HR-pQCT-determined vBMD estimates at the radius and tibia. Although these imaging modalities had poor agreement, total, trabecular, and cortical vBMD and surrogate measures of bone strength were strongly correlated at all skeletal sites.

Our precision estimates for DXA and pQCT variables were consistent with previous literature.^7–12^ CVs for most HR-pQCT bone variables derived from scans that underwent 3DR were generally lower compared with those that underwent 2DR. At both the radius and tibia, most of the bone variables we assessed at fixed sites, and all variables at the proximal 30% sites, had lower CVs following 3DR. CVs for most bone variables at the 4% site of the radius improved with 3DR; however, only half of the bone variables at the 4% site of the tibia improved. The more consistent precision improvements at the radius than the tibia following 3DR may be attributed to the fact that positioning of the forearm is more difficult compared with the lower leg. Given 3DR additionally accounts for rotational misalignment, this technique is likely to outperform 2DR at skeletal sites where participant positioning is most difficult. Similarly, Kemp et al.^20^ showed that 3DR led to improved reproducibility of total vBMD estimates at the radius, but not at the tibia, compared with 2DR. Ellouz et al.^19^ reported no improvements in the precision of densitometric estimates at the radius or tibia with 3DR compared with 2DR; however, improvements were noted for geometric outcomes and measures of cortical structure at both skeletal sites.

HR-pQCT-derived cortical porosity had poor reproducibility at all skeletal sites, irrespective of the registration method used. Chiba et al. and Ellouz et al. improved cortical porosity precision to a greater degree than we did in the current study, and all their CVs were below 6.7% using 3DR.^16^^,^^19^ We did not observe cortical porosity CVs below 11% at any skeletal site using 3DR, which could be related to differences in 3DR methods used, scanner generation, target populations, or scanning protocols.^16^^,^^19^ We used the same 3DR technique as Kemp et al.,^20^ who also reported minimal or no improvement with 3DR compared with 2DR. Poor reproducibility of cortical porosity estimates could be partly related to age with Burghardt et al.^27^ showing that cortical porosity CVs at the tibia were significantly better in an older cohort (aged >40 yr) compared with a younger cohort (aged <40 yr). Similar findings have been reported elsewhere.^15^ Another factor that explains the poor reproducibility of this measure is the resolution of HR-pQCT scanners. Haversian canal diameters can be as small as 20-30 μm, meaning some are too small to detect via HR-pQCT (voxel size approximately 60 μm). Although the number of pores with diameters smaller than 90 μm is relatively high (>60%), they only contribute approximately 5%-8% of the total pore volume.^28^ Ultimately, 3DR appears to either outperform or be as effective as 2DR in improving the reproducibility of most bone variables. As such, this technique is important to implement in longitudinal studies, especially those involving bone-targeted interventions such as antiresorptive or exercise therapy.

Densitometric estimates from pQCT and HR-pQCT had poor agreement, with pQCT consistently providing higher vBMD estimates. Total, trabecular and cortical vBMD differed by 9%-50% at the radius and 14%-32% at the tibia. This finding was unsurprising given pQCT and HR-pQCT assign different density values to tissues.^4^ Therefore, cross-calibration is required to directly compare densitometric estimates between these imaging modalities. An ex vivo study of 15 human tibias reported an average difference of approximately 15% and 17% for cortical and trabecular vBMD, respectively.^5^^,^^29^ Similarly, in 16 adults, Hildebrand et al.^6^ reported 18% lower HR-pQCT-estimated cortical vBMD compared with pQCT. Our study found better agreement between pQCT and HR-pQCT for cortical vBMD estimates, but poorer agreement for total and trabecular vBMD (approximately 22%-26%) at the radius and tibia. The lowest agreement we observed was for comparisons between pQCT-derived trabecular vBMD and HR-pQCT-derived inner trabecular vBMD. We compared these variables given pQCT defines the inner 45% of the CSA as trabecular bone, and HR-pQCT estimates inner trabecular vBMD within the inner 60% of the trabecular bone CSA. It is unclear why these variables had the poorest agreement. Other factors that explain differences in densitometric estimates between these imaging modalities include both being affected to different degrees by the partial volume effect, which is related to spatial resolution, as well as differences in scan lengths, which resulted in slightly different areas of the bone being assessed (with overlapping regions of interest).^4^

We observed strong correlations between pQCT- and HR-pQCT-derived bone density and strength estimates (failure load and stiffness), suggesting these imaging modalities have similar fracture prediction capabilities in healthy older adults. This finding is important given the advantages pQCT has over HR-pQCT with respect to cost and portability, making it an attractive alternative in low-resource settings. Nevertheless, the poorer resolution of pQCT relative to HR-pQCT results in partial volume effects (leading to poorer precision/reproducibility) and the inability to directly assess bone microarchitecture, which predicts fractures independently of aBMD.^30–32^ HR-pQCT-determined total and trabecular vBMD has excellent precision at the tibia and radius (CVs < 0.85%), and these bone variables are the most reliable predictors of fracture.^14^ Although bone strength estimates are also predictive of fracture, their poorer precision makes them less reliable compared with densitometric estimates.^14^^,^^33^

This study had some limitations. We recruited adults aged >50 yr, and no participants were excluded based on their bone health status, so our precision estimates may differ from other populations with better or worse bone health.^15^ Motion artifact is a source of error during in vivo HR-pQCT image acquisition, and increased participant movements introduce artefactual errors that cannot be corrected through registration techniques.^34^ However, scans with significant motion artifact were excluded from our analyses. Some of our pQCT and HR-pQCT precision estimates may have been underpowered following the omission of poor-quality scans. Precision estimates for FEA were not calculated using 3DR due to the complex proximal and distal surfaces of the common region identified using this technique. We scanned slightly different proximal sites using pQCT and HR-pQCT, which may have contributed to poor agreement between densitometric variables. Unfortunately, HR-pQCT scans could not be performed at more proximal sites due to the design of manufacturers’ arm and leg casts. Scan lengths also differed between pQCT and HR-pQCT, however, obtaining three contiguous 2.5 mm slices using pQCT has been shown to have no difference compared to a single 2.5 mm slice.^35^ Nevertheless, the volumes of bone we compared were not identical.

In conclusion, we found low precision errors within the expected range for all imaging modalities. 3D registration improved precision for most bone variables assessed via HR-pQCT. Systematic bias was identified between pQCT- and HR-pQCT-determined radial and tibial vBMD, emphasizing the need for cautious interpretation when comparing results between these modalities. Despite this, we observed strong correlations between densitometric variables assessed by both imaging modalities, suggesting they have similar fracture prediction capabilities. Future longitudinal studies involving HR-pQCT-derived bone outcomes should utilize 3DR to improve reproducibility when monitoring changes in different bone compartments.

## Supplementary Material

Supplementary_data_JBMRPlus_ziae158

## Data Availability

The data that support the findings of this study are available from the corresponding author, upon reasonable request.
