# Supplementary material for: Bone imaging modality precision and agreement between DXA, pQCT, and HR-pQCT
Source: JBMR Plus. 2024 Dec 3;9(2):ziae158. doi: 10.1093/jbmrpl/ziae158 (PMC11752644; doi:10.1093/jbmrpl/ziae158)
Supplement: Supplementary_data_JBMRPlus_ziae158 [file supplementary_data_jbmrplus_ziae158.docx]

**Supplementary data**

**Table S1**. Pearson correlations between various bone strength estimates from pQCT and HRpQCT at the proximal radius and tibia.

|  | **HR-pQCT (30%)** | **pQCT** | **Correlation Coefficient (95%CI)** |
| --- | --- | --- | --- |
| **Radius (33%)** | Failure Load (N) | SSI Polar (mm^3^) | -0.914 (-0.961, -0.816) |
|  |  | pMOIa (mm^4^) | -0.926 (-0.967, -0.841) |
|  |  | pMOIw (mm^4^) | -0.940 (-0.973, -0.868) |
|  | pMOI (mm^4^) | SSI Polar (mm^3^) | 0.650 (0.352, 0.829) |
|  |  | pMOIa (mm^4^) | 0.547 (0.203, 0.771) |
|  |  | pMOIw (mm^4^) | 0.587 (0.258, 0.794) |
|  | Stiffness (N/mm) | SSI Polar (mm^3^) | 0.924 (0.837, 0.966) |
|  |  | pMOIa (mm^4^) | 0.936 (0.860, 0.971) |
|  |  | pMOIw (mm^4^) | 0.948 (0.885, 0.977) |
| **Tibia (38%)** | Failure Load (N) | SSI Polar (mm^3^) | -0.926 (-0.965, -0.846) |
|  |  | pMOIa (mm^4^) | -0.900 (-0.952, -0.795) |
|  |  | pMOIw (mm^4^) | -0.925 (-0.964, -0.845) |
|  | pMOI (mm^4^) | SSI Polar (mm^3^) | 0.626 (0.337, 0.808) |
|  |  | pMOIa (mm^4^) | 0.520 (0.190, 0.745) |
|  |  | pMOIw (mm^4^) | 0.585 (0.278, 0.784) |
|  | Stiffness (N/mm) | SSI Polar (mm^3^) | 0.932 (0.860, 0.968) |
|  |  | pMOIa (mm^4^) | 0.905 (0.806, 0.955) |
|  |  | pMOIw (mm^4^) | 0.930 (0.856, 0.967) |

pMOI – polar moment of inertia; pMOIa - polar moment of inertia based on cortical area; pMOIw - polar moment of inertia based on cortical area weighted by cortical density.
